# Supplementary figures and images for: Distinct patterns of SARS-CoV-2 BA.2.87.1 and JN.1 variants in immune evasion, antigenicity, and cell-cell fusion
Source: mBio. 2024 Apr 9;15(5):e00751-24. doi: 10.1128/mbio.00751-24 (PMC11077997; doi:10.1128/mbio.00751-24)

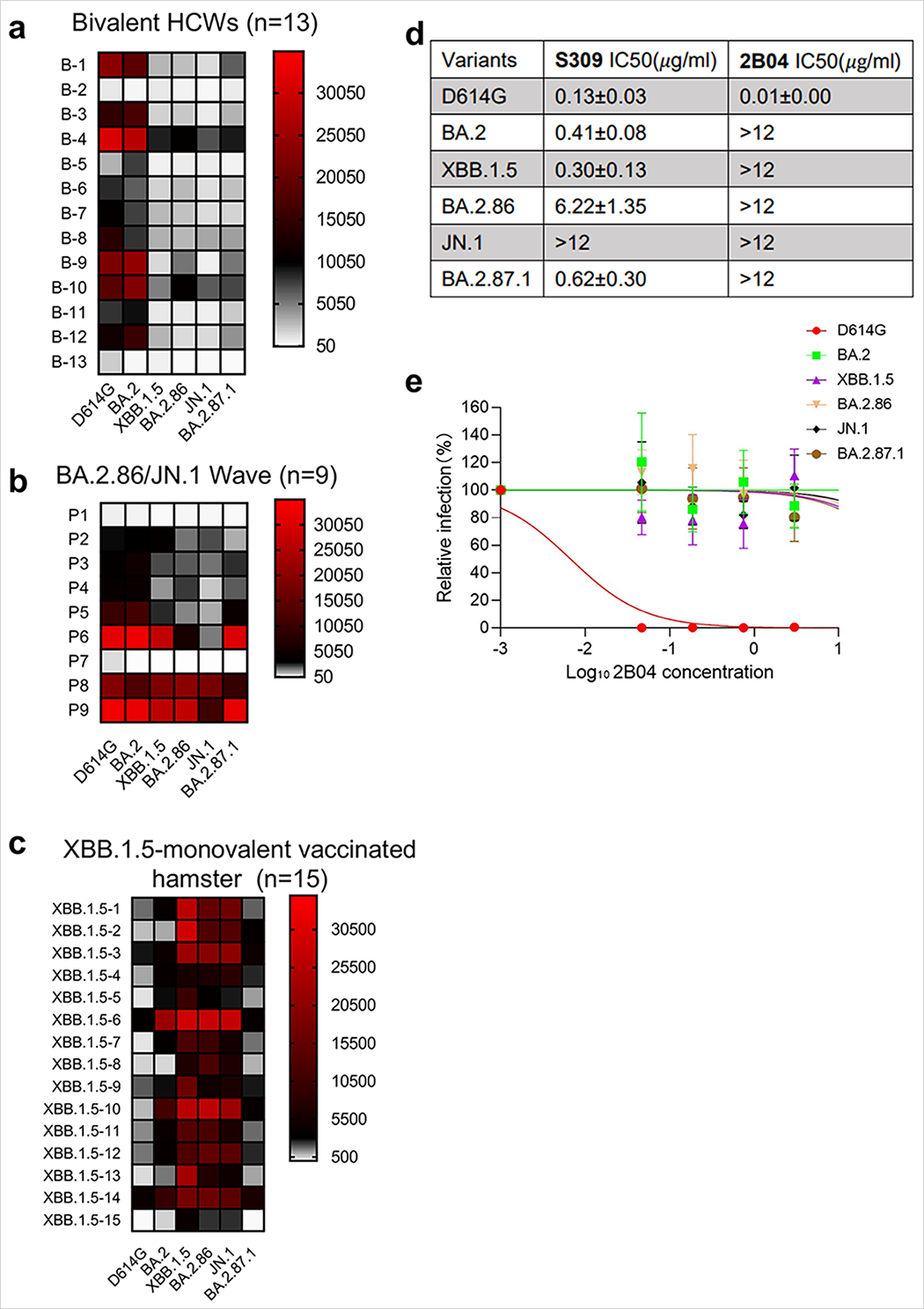

Supplement: Fig. S1 — Heatmap display of nAb escape in three cohorts. [file mbio.00751-24-s0001.tif]

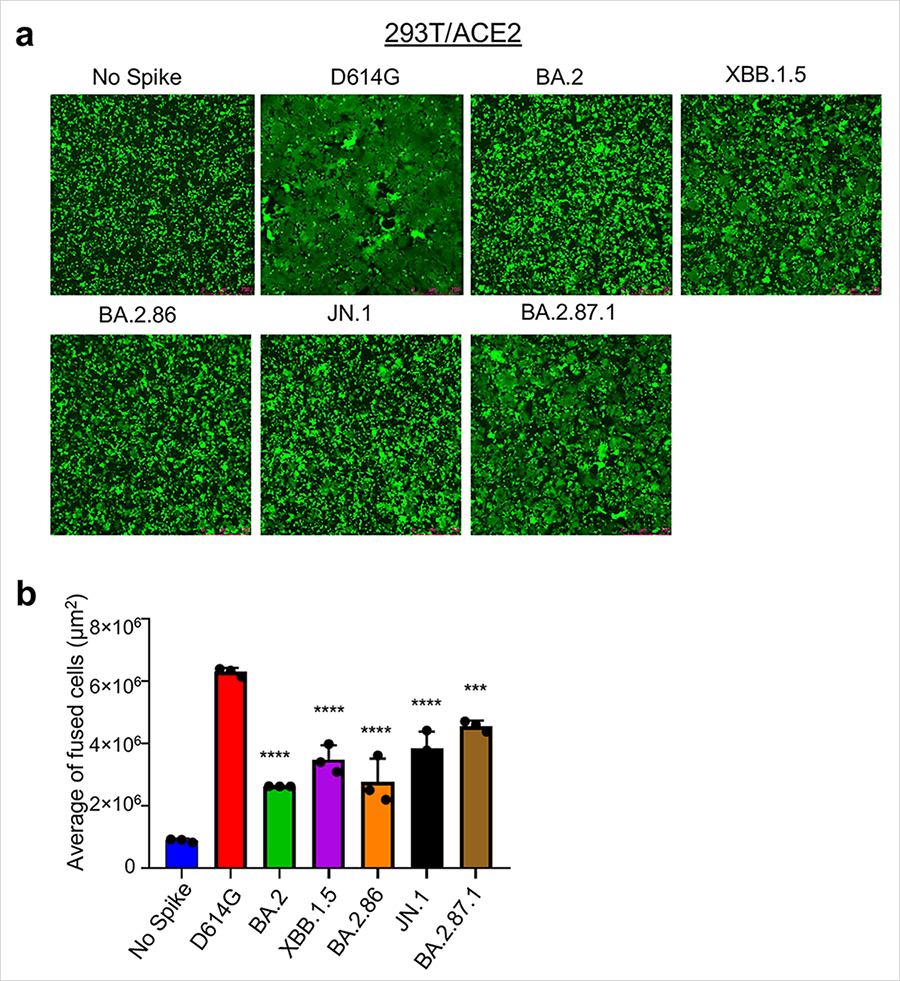

Supplement: Fig. S2 — Syncytium formation induced by BA.2.87.1, JN.1 or other Omicron spike proteins. [file mbio.00751-24-s0002.tif]
