# Supplementary material for: Distinct patterns of SARS-CoV-2 BA.2.87.1 and JN.1 variants in immune evasion, antigenicity, and cell-cell fusion
Source: mBio. 2024 Apr 9;15(5):e00751-24. doi: 10.1128/mbio.00751-24 (PMC11077997; doi:10.1128/mbio.00751-24)
Supplement: Table S1 — Bivalent-vaccinated HCW and BA.2.86/JN.1-wave first responder cohorts. [file mbio.00751-24-s0004.docx]

**Table S1. Bivalent-vaccinated HCW and BA.2.86/JN.1-wave first responder cohorts**

|  | **Bivalent HCWs**  **(n=13)** | BA.2.86-JN.1 Wave Patients  (n=9) |
| --- | --- | --- |
|  |  |  |
| **Age in Years at Sample** Collection  [Median (Range)] | 37 (25-48) | 53(35-78) |
| Gender [n (% of Total)] |  |  |
| Male | 8 (61.5%) | 4 (44.4%) |
| Female | 5 (38.5%) | 5 (55.6%) |
| Sample Collection Window | Dec. 2022- Jan.2023 | Nov. 2023-Feb.2024 |
| Vaccine status [n (% of Total)] |  |  |
|  |  |  |
| 2-dose Moderna | NA | 3 (11.1%) |
| 1-dose Moderna+1-dose Pfizer bivalent | NA | 1 (11.1%) |
| 3-dose Pfizer +1-dose Moderna bivalent | 1 (7.7%) | 0 |
| 2-dose Pfizer+1-dose Pfizer bivalent | 1 (7.7%) | 0 |
| 4-dose Pfizer+1-dose Pfizer bivalent | 1 (7.7%) | 0 |
| 3-dose Pfizer+1-dose Pfizer bivalent | 3 (23.1%) | 0 |
| 3-dose Moderna+1-dose Moderna bivalent | 6 (46.2%) | 1 (11.1%) |
| 2-dose Moderna+1 Pfizer+1-dose Pfizer bivalent | 1 (7.7%) | 0 |
| 3-dose Moderna+1-dose Pfizer bivalent+1-dose Moderna bivalent | 0 | 1 (11.1%) |
| 2-dose Pfizer +1-dose Moderna+1-dose Moderna bivalent | 0 | 1 (11.1%) |
| 1-dose Pfizer | 0 | 1 (11.1%) |
| 3-dose Moderana+1-dose XBB.1.5 Moderna monovalent | 0 | 1 (11.1%) |
| Sample Collection Timing [Median (Range)] |  |  |
| Days from last vaccination | NA | 656 (45-898) |
| Days post the bivalent dose for recipients | 66 (23-108) | NA |
| COVID-19 positive [n (% of Total)] | 9 (69.2%) | 9 (100%) |
| Days before sample collection [Median (Range)] | 324 (182-994) | 7 (1-10) |
| Infected Variants |  |  |
| JN.1/BA.2.86 | 0 | 2 (22.2%) |
| Undetermined | NA | 7 (77.8%) |

Summary of the demographic information for two cohorts used for neutralization experiments depicted in Figure 2. “NA” means the category is not applicable to the cohort.
